# Prevalence of Gastroesophageal Reflux Disease in Type II Diabetes Mellitus

**DOI:** 10.1155/2014/601571

**Published:** 2014-10-29

**Authors:** Huihui Sun, Lisha Yi, Ping Wu, Yingjie Li, Bin Luo, Shuchang Xu

**Affiliations:** ^1^Department of Gastroenterology, Tongji Institute of Digestive Diseases, Tongji Hospital, Tongji University, No. 389 Xin Cun Road, Shanghai 200065, China; ^2^Department of Clinical Nutrition, Tongji Hospital, Tongji University, No. 389 Xin Cun Road, Shanghai 200065, China

## Abstract

*Background/Aims*. Patients with type II diabetes mellitus (DM) were known to have higher prevalence of gastroesophageal reflux disease (GERD) in the Western countries, but data on the impact of GERD on DM patients in our country are scarce. The aim of this study was to evaluate the prevalence of GERD in type II DM patients in Shanghai, China, and to explore its possible risk factors. *Methods*. 775 type II DM cases were randomly collected. Reflux Disease Questionnaire (RDQ) was used to check the presence of GERD. Patients' characteristics, laboratory data, face-to-face interview, nerve conduction study, and needle electromyogram (EMG) test were analyzed. *Results*. 16% patients were found with typical GERD symptoms. Pathophysiological factors such as peripheral neuropathy, metabolism syndrome, and obesity were found to have no significant differences between GERD and non-GERD type II DM patients in the present study. *Conclusion*. The prevalence of GERD in type II DM patients is higher than that in adult inhabitants in Shanghai, China. No difference in pathophysiological factors, such as peripheral neuropathy, and metabolism syndrome was found in DM-GERD patients, suggesting that further study and efforts are needed to explore deeper the potential risk factors for the high prevalence rate of GERD in DM patients.

## 1. Introduction

Gastroesophageal reflux disease (GERD) is defined as “symptoms or complications associated with regurgitation from the stomach and (or) the duodenum to the esophagus.” It is associated with Barrett's esophagus and esophageal carcinogenesis. Its typical symptoms include heartburn and sour regurgitation. The prevalence of GERD has been increasing worldwide and seriously influences the living quality of patients such as sleep dysfunction, metabolic disorders, and heart disease. Many studies have shown that diabetes mellitus (DM) patients are often with high incidence of GERD symptoms. In 2008, Wang et al. studied 150 type II DM patients in USA and found that the overall prevalence of GERD symptoms in diabetics is 40.7% [1]. In 2012, Hirata et al. investigated 66 Japanese type II DM outpatients by Frequency Scale for the Symptoms of GERD (FSSG), and their results showed that the prevalence of GERD symptoms in type II DM was 23% [2]. In addition, one Korean study reported that 18% erosive esophagitis among patients with DM who undergone esophagogastroduodenoscopy (EGD) was due to various GI symptoms [3]. However, the exact prevalence of GERD in patients with type II DM in our country is still not clear.

Up to present, the pathogenesis of GERD in type II DM patients has not been fully clarified. In previous studies, many pathophysiological factors such as obesity, peripheral neuropathy, metabolism syndrome, and FBG were thought as possible risk factors for the high prevalence of the typical GERD symptoms in type II DM patients. Peripheral neuropathy especially has become a research hotspot in recent years [1, 4]. However, the impact of the above factors on the presence of GERD symptoms in type II DM patients is still under debate. For example, DM patients with neuropathy were reported to have significantly more GI symptoms than those without neuropathy in many studies [1, 5, 6]. In contrast to these reports, in 1989, Clouse and Lustman reported that gastrointestinal symptoms occurring in diabetic patients were poorly related to neuropathic complications [7]. In 2011, Lee et al. showed that there was no significant difference in the proportions of patients experiencing typical GERD symptoms between the 2 groups of type II DM with and without neuropathy [4]. The pathogenesis for the more presence of GERD symptoms in patients with type II DM needs more exploration.

Our study was done to evaluate the prevalence of GERD symptoms in type II DM patients in Shanghai, China, and to explore whether peripheral neuropathy and other factors such as obesity and metabolism syndrome are the possible contributory factors for the pathogenesis of GERD in type II DM, attempting to clarify the pathogenesis for the presence of GERD in type II DM in our country.

## 2. Materials and Methods

### 2.1. Study Population

The study protocol was approved by the Tongji Hospital Ethics Committee. All subjects signed an informed consent form before the study started. Baseline characteristics (sex, age, height, weight, body mass index (BMI), waist circumference (WC), hip circumference (HC), and waist-to-hip ratio (WHR)), laboratory parameters (fasting blood-glucose (FBG), triglyceride (TG), high density lipoprotein (HDL), total cholesterol (TC), and low high density lipoprotein (LDL)), interview indicators (smoking, drinking, DM family history, hypertension, metabolism syndrome, and treatment with insulin), and test results of nerve conduction and needle EMG were observed and analyzed. All patients with type II DM underwent 2 steps of investigations: (a) Reflux Disease Questionnaire (RDQ) and (b) face-to-face interview indicators.

### 2.2. Diagnosis of Diabetes Mellitus Neuropathy

Patients underwent nerve conduction study and needle EMG for the evaluation of neuropathy. The latency, amplitude, and nerve conduction velocity of motor and sensory nerves were recorded on the median and ulnar nerves of upper extremities and peroneal, posterior tibial, and sural nerves of lower extremities. Peripheral neuropathy was defined as at least 2 different nerves were found to be abnormal in decreased amplitude or delayed nerve conduction velocity.

### 2.3. Diagnosis of Gastroesophageal Reflux Disease

RDQ comprises 8 questions assessing the frequency and severity of heartburn, substernal chest pain, acid regurgitation, and food reflux in the past 4 weeks which are scored on a 5-point Likert scale. Each symptom was scored according to the severity and frequency (five scales). The highest score for one patient was 40. Symptom-based diagnosis of GERD was made when a patient had a score more than 12 [8].

### 2.4. Statistical Methods

Statistical analysis was performed using SPSS17.0 software. Continuous variables were tested with Student's *t*-test and categorical variables were tested with Chi-square test. *P* < 0.05 was selected as a significant level.

## 3. Results

### 3.1. Baseline Characteristics of the Subjects in Each Group

In our study, 775 type II DM patients were randomly selected from the outpatient department of Shanghai Tongji Hospital between April 2013 and April 2014. After RDQ questionnaire, 124 type II DM patients were found with GERD (the prevalence rate of GERD is 16%). According to the results of RDQ questionnaire, 775 DM patients were divided into two groups: GERD group and non-GERD group. Baseline characteristics of two groups were shown in Table 1. No significant differences were found between the above two groups with regard to sex composition, age, height, weight, BMI, WC, HC, and WHR.

### 3.2. Comparison of Laboratory Parameters between the Two Groups

Laboratory parameters including FBG, TG, TC, LDL, and HDL were compared between GERD and non-GERD groups. There were no significant differences between the two groups (see Table 2).

### 3.3. Comparison of Face-to-Face Interview Indicators between the Two Groups

Face-to-face interview data such as smoking, drinking, DM family history, hypertension, metabolism syndrome, and treatment with insulin were compared between GERD and non-GERD groups. No factors were found to have significant differences between the two groups (see Table 3).

### 3.4. Comparison of Neuropathy between the Two Groups

After nerve conduction study and needle EMG, 461 type II DM patients were found with peripheral neuropathy (the prevalence rate of peripheral neuropathy is 59%). Compared between GERD and non-GERD groups, the prevalence rate of peripheral neuropathy has no significant difference (see Table 3).

## 4. Discussion

The prevalence of GERD in type II DM patients is higher than that in adult inhabitants in Shanghai, China. Some pathophysiological factors, such as peripheral neuropathy, metabolism syndrome, and obesity, have no significant difference between GERD-DM and non-GERD-DM patients.

Type II DM has been described as a possible risk factor for the development of GERD. One USA study reported that the prevalence of GERD symptoms in type II DM patients was approximately 41% [1]. Some studies on the Japanese or Korean indicated that the prevalence of GERD symptoms in patients with type II DM was approximately 18–25% [2, 3, 5, 9, 10]. GERD is a relatively common disorder in the Western populations, and approximately 10–20% of adults were reported to experience heartburn and/or acid regurgitation at least once a week [11]. The prevalence of GERD is relatively lower in Asian populations (approximately 5%) [12, 13] but is reported to be increasing. All previous studies indicted that the prevalence of GERD symptoms in patients with type II DM was higher than the general population. In the present study, the prevalence of GERD diagnosed by RDQ > 12 in type II DM patients was 16%, which was higher than that in adult inhabitants in Shanghai, China. In 2009, Ma et al. [14] and Wang et al. [15] through investigating adult inhabitants of Shanghai found that the prevalence of GERD was approximately 6.2% in Shanghai, and GERD has a serious impact on health-related quality of life of Shanghai inhabitants. In a word, the prevalence of GERD symptoms in patients with type II DM is higher than generation populations in Shanghai, China.

In previous studies, pathophysiological factors such as obesity, FBG, TG, HDL, hypertension, metabolism syndrome, insulin, and peripheral neuropathy were all thought of as possible risk factors for the high prevalence of the typical GERD symptoms in DM patients [2, 5, 16, 17]. Particularly, neuropathy, the high prevalence of GI symptoms in patients with DM was reported to be possibly associated with its neuropathy in many studies [1, 4]. For example, in 2008, Wang et al. [1] reported that the prevalence of GERD symptoms was higher in patients with neuropathy than in patients without neuropathy. The prevalence of heartburn, chest pain, and chronic cough is also higher in patients with neuropathy than in patients without neuropathy. In contrast to these reports, the present study showed that the proportions of neuropathy had no significant difference between DM patients with and without GERD symptoms, suggesting that the prevalence of GERD symptoms in type II DM patients with neuropathy has no significant difference with that in type II DM patients without neuropathy. Our results are similar to the studies of Clouse and Lustman [7] and Lee et al. [4]. In 1989, Clouse and Lustman [7], through analyzing 114 diabetic subjects with gastrointestinal motor dysfunction symptoms, reported that gastrointestinal symptoms occurring in diabetic patients were poorly related to neuropathic complications. In 2011, Lee et al. [4] studied 119 patients with type II DM and found that there was no significant difference in the proportions of patients experiencing typical GERD symptoms between the 2 groups of type II DM with and without neuropathy. More research is required to explore the impact of neuropathy on the presence of GERD symptoms in patients with type II DM.

It was reported that metabolic syndrome was associated with the higher prevalence of GERD symptom in subjects with type II DM [2]. In some studies, it has been mentioned that factors like obesity [2, 18, 19], hypertension, dyslipidemia [2], poor glycemic control [17], insulin [16], and so forth may be contributory factors for the presence of GERD symptom in type II DM patients. However, some other studies reveal that this may not be the case [1]. In the present study, we also found that BMI, WC, HC, WHR, FBG, TG, TC, LDL, HDL, hypertension, and treatment with insulin had no significant differences between GERD group and non-GERD group in DM patients. In addition, smoking, drinking, and DM family history were also explored in our study, and they also had no significant differences between the above two groups.

In recent years, the effect of psychiatric factors was gradually focused. Some previous studies have reported that gastrointestinal symptoms in diabetic patients may be poorly related to DM complication; instead they were related to psychiatric factors [7, 20]. In present study, we also found that pathophysiological factors such as peripheral neuropathy and metabolism syndrome all had no significant differences between GERD and non-GERD groups in type II DM patients. So, we speculate that the high prevalence of GERD symptoms in patients with type II DM may be due to psychiatric factors, but not pathophysiological factors. However, due to the limited subjects in this research, the effect of pathophysiological factors cannot be fully eliminated. Further investigation with more subjects will be performed as the next-step research.

In summary, the prevalence of GERD in type II DM patients is higher than that in adult inhabitants in Shanghai, China. We speculate that the high prevalence of GERD symptoms in patients with type II DM may be due to psychiatric factors, but not pathophysiological factors.

## Figures and Tables

**Table 1:** Baseline characteristics of GERD group and non-GERD group.

Demographics parameters	GERD (*n* = 124)	Non-GERD (*n* = 651)	*P *
Female patients, *n* (%)	75 (60.5%)	346 (53.1%)	0.080
Age (mean ± SD), years	62.70 ± 14.01	65.33 ± 13.62	0.051
Duration of diabetes, years	10.60 ± 8.31	11.40 ± 7.76	0.205
Height (mean ± SD), cm	163.05 ± 8.10	164.31 ± 8.51	0.129
Weight (mean ± SD), kg	64.32 ± 12.62	66.10 ± 11.92	0.130
BMI (mean ± SD), kg/m^2^	24.09 ± 3.68	24.39 ± 3.84	0.417
WC (mean ± SD), cm	91.08 ± 10.44	89.91 ± 10.88	0.267
HC (mean ± SD), cm	94.40 ± 8.76	93.70 ± 8.93	0.432
WHR (mean ± SD)	0.96 ± 0.09	0.97 ± 0.08	0.104

BMI: body mass index; WC: waist circumference; HC: hip circumference; WHR: waist-to-hip ratio; *P* < 0.05 was selected as a significant level.

**Table 2:** Comparison of laboratory data between GERD group and non-GERD group.

	GERD (*n* = 124)	Non-GERD (*n* = 651)	*P *
FBG (mean ± SD), mmol/L	8.94 ± 3.71	8.33 ± 3.26	0.072
TG (mean ± SD), mmol/L	1.82 ± 1.23	1.83 ± 1.49	0.969
TC (mean ± SD), mmol/L	4.58 ± 1.21	4.65 ± 1.22	0.590
LDL (mean ± SD), mmol/L	2.57 ± 0.90	2.64 ± 0.92	0.429
HDL (mean ± SD), mmol/L	1.07 ± 0.32	1.10 ± 0.31	0.300

FBG: fasting blood glucose; TG: triglyceride; TC: total cholesterol; LDL: low density lipoprotein; HDL: high density lipoprotein; *P* < 0.05 was selected as a significant level.

**Table 3:** Comparison of interview indicators and neuropathy between the two groups.

	GERD (*n* = 124)	Non-GERD (*n* = 651)	*χ* ^2^	*P *
Smoking (+/−)	24/100	133/518	0.903	0.446
Drinking (+/−)	13/111	52/599	0.376	0.224
DM family history (+/−)	36/88	165/486	0.434	0.226
Hypertension (+/−)	96/28	505/146	1.000	0.526
Metabolism syndrome (+/−)	84/40	409/242	0.310	0.174
Treatment with insulin (+/−)	98/26	511/140	1.000	0.500
Neuropathy (+/−)	71/53	390/261	0.618	0.325

*P* < 0.05 was selected as a significant level.

## Abbreviations

GERD: Gastroesophageal reflux disease
DM: Diabetes mellitus
FSSG: Frequency scale for the symptoms of GERD
EGD: Esophagogastroduodenoscopy
BMI: Body mass index
WC: Waist circumference
HC: Hip circumference
FBG: Fasting blood glucose
TG: Triglyceride
TC: Total cholesterol
LDL: Low density lipoprotein
HDL: High density lipoprotein
RDQ: Reflux Disease Questionnaire.
